# Healthcare utilization and associated factors during the first wave of COVID‐19 pandemic in Ghana: A cross‐sectional survey

**DOI:** 10.1002/hsr2.1805

**Published:** 2024-01-10

**Authors:** Dominic D. Gadeka, Justice M. K. Aheto

**Affiliations:** ^1^ Department of Health Policy, Planning and Management University of Ghana School of Public Health Legon‐Accra Ghana; ^2^ Department of Biostatistics University of Ghana School of Public Health Legon‐Accra Ghana; ^3^ WorldPop, School of Geography and Environmental Science University of Southampton Southampton UK; ^4^ College of Public Health University of South Florida Tampa Florida USA

**Keywords:** COVID‐19, Ghana, healthcare utilization, health insurance, pandemic, risk factors

## Abstract

**Background and Aims:**

Understanding healthcare utilization during the coronavirus disease 2019 (COVID‐19) pandemic is crucial to inform policy and to prepare health systems for future pandemics. We examined self‐reported healthcare utilization and associated factors, including public health preventive practices, perceptions, and coping strategies among the general public in Ghana during the first wave of the COVID‐19 pandemic.

**Methods:**

We adopted a cross‐sectional study design using a public survey to recruit 643 respondents between May 23, and July 11, 2020 during the first wave of confirmed COVID‐19 cases and after the fifth week of a partial lockdown in Ghana. Descriptive, bivariate, and binary logistic regression analyses were carried out in Stata version 15.

**Results:**

Overall, there was a high level of compliance with COVID‐19 public health preventive measures. In terms of perception, 357 (55.5%) of respondents stated unnecessary worry was created about the disease. In relation to coping strategies, 376 (58.5%) of respondents stayed home for more than 6 h, while 35 (5%) reported drinking alcohol to overcome the fear created by the disease. The results showed that 176 (27.4%) of the respondents utilized healthcare while 44 (9.4%) did not utilized healthcare for fear of contracting the disease at the health facility. Marital status (adjusted odds ratio [aOR] = 0.63; 95% confidence interval [CI] = 0.409, 0.963), religion (aOR = 2.34; 95% CI = 1.10, 4.98), and possession of valid health insurance (aOR = 1.51; 95% CI = 1.020, 2.235) were associated with healthcare utilization.

**Conclusion:**

There was low healthcare utilization coupled with fear of contracting the COVID‐19 disease at the health facilities among the respondents. The findings suggest the need for effective public education that ensures that future pandemics' prevention information and recommendations are easily understandable by the general public. Additionally, investment in health insurance coverage may contribute to healthcare utilization during future pandemics.

## 1 BACKGROUND

On March 11, 2020, the World Health Organization (WHO) declared the coronavirus disease 2019 (COVID‐19) disease a global pandemic.^1^ By the end of March 2020, most countries across the world had put in place major preventive measures like lockdowns and containment policies, including the closure of schools, curfews, restricted movements, travel bans, and many others to contain the spread of the disease.^2^ In the absence of definitive curative measures to prevent the transmission of the disease, public health‐preventative measures including social distancing, wearing of face masks, washing of hands with soap under running water and use of hand sanitizer became the surest means of mitigating the disease.^2^, ^3^

By the end of the first wave of the pandemic, many affected countries reported significant effects across all functions of the healthcare system and decline in healthcare utilization.^4^, ^5^, ^6^, ^7^ In high‐income countries, lockdown policies including curfews, “stay‐at‐home” and suspension of routine healthcare to provide room for COVID‐19 patients as well as social distancing measures and fear of contracting the virus within health facilities were attributed to the decline.^2^, ^6^ Many patients were reported to defer or forego routine healthcare, especially elective and preventive healthcare visits^5^ while several others were discouraged from attending hospitals by their usual healthcare providers.^6^, ^7^, ^8^ However, in low‐and‐middle‐income countries, there has been limited evidence on the rate of utilization of healthcare, particularly during the first wave of the pandemic. Moreover, factors that were found to have facilitated or mitigated the utilization during this phase of the pandemic is limited.

In Ghana, the first two cases of the COVID‐19 disease were recorded on March 12, 2020. Following that, a partial lockdown was announced and implemented in two major cities (Greater Accra and Greater Kumasi) considered as epicenters of the disease, from 01:00 (local time) beginning March 30, 2020 to April 19, 2020. Citizens were only permitted to leave their homes for essential items including food, medicine, water, pay their utility bills, as well as visit the hospital, pharmacies or bank while observing the WHO's COVID‐19 prevention protocols.^9^, ^10^ By the end of the 3 weeks of the partial lockdown, 1042 cases were confirmed, stretching the healthcare system. As a result, many people were allowed to isolate and be treated in their homes.^9^, ^10^ Misconceptions about the disease also grew within same period, while stigma on survivors of COVID‐19 deepened.

While these could affect healthcare utilization among the general public, available evidence is scanty. However, low healthcare utilization during a pandemic of this nature is likely to reverse decades of progress in improving health outcomes. This could further put people at increased risk of avoidable illness and death.

This study examined healthcare utilization among the general public and associated factors during the first wave of the COVID‐19 pandemic in Ghana to offer insights and to enable plan for compensating for missed healthcare opportunities.

## 2 METHODS

### 2.1 Study design

This was a cross‐sectional study design involving a population‐based online survey. The approach enabled the capture of adequate data needed within a short time during the first wave between May 23, and July 11, 2020 after the fifth week of a partial lockdown in two major cities (Greater Accra and Greater Kumasi) considered as epicenters of the disease in Ghana. Kasoa which is part of the Central region but shared boundary with Accra was also placed under the lockdown. The study examined the healthcare utilization and sociodemographic predictors as well as attitude and behavioral practices during the COVID‐19 pandemic in Ghana among the general public.

### 2.2 Data collection tool

A structured self‐reported questionnaire was used. The questionnaire was created using Google form and administered in English (https://docs.google.com/forms/). The Google form consisted of a “Participant Information Sheet” containing brief information about the objectives, risks, and benefits, expected duration of participation, voluntary nature of participation and declaration of confidentiality. Confidentiality and anonymity of all participants were maintained.

The first page of the questionnaire explained that their participation was completely voluntary, and no coercion or inducement would be applied to get them involved. Furthermore, the first page explained that the participants had the right to withdraw from the study at any point without justifying or explaining to the researcher the reason and withdrawal would not in any way attract any sanction. A compulsory field was created for the participants to agree or to decline participation in the study and only those who agreed to participate had access to the main questionnaire to complete.

The first section of the questionnaire consisted of sociodemographic information including age, sex, education, marital status, employment status, religion of respondent, nationality, average earnings per month and household size while the second section included, precautionary measures against COVID‐19, social attitude and adaptive coping strategies and health service utilization by the general public. Precautionary measures included covering of mouth when coughing or sneezing, avoiding sharing of utensils, washing of hands under running water with soap, sanitizing of hands with alcohol‐based sanitizer frequently, wearing of face mask to public places and washing or sanitizing hands after touching contaminated objects/surfaces.

The section on social attitude and adaptive coping included average number of hours respondent stayed at home to avoid COVID‐19, drinking of alcohol to get through the fear/anxiety caused by the COVID‐19 disease, whether respondent takes medication to overcome the fear created by the disease, and whether there was an unnecessary worry made about the COVID‐19. Questions on health service utilization were whether the respondent ever visited any hospital or health facility for healthcare in the last weeks of COVID‐19 in Ghana, whether respondents received fair treatment when they visited the health facility for care, whether respondent was satisfied with the health service provided, why respondent never visited health facility and whether respondent had valid insurance cover (medical insurance that has not expired).

### 2.3 Data collection and sampling

A convenience sampling approach was used to recruit the respondents. Before the distribution of the study questionnaire, the online survey was piloted by the research team, with amendments made to ensure the questions were explicit and clear. The link to the online survey was shared virtually through advertising on social media platforms, specifically WhatsApp and Facebook platforms. Network of colleagues' personal contacts were also employed to promote the survey. Weekly reminders were sent to further enhance the response rate.^10^ The security features of the online portal were designed to allow single participation only. The survey tool automatically verified that all questions had to be filled completely before submission. Once an online questionnaire was completed, it was automatically sent to the survey platform hosted by the first author for collation. To ensure anonymity and protection of rights, participants' identities were not sought. Participants were automatically assigned codes in Google Forms, rather than the use of personal identification details. The questionnaire took about 10–15 min to be completed. Data obtained were encrypted with a password for safety and confidentiality.

### 2.4 Data analysis

Responses received through the Google form were obtained as excel sheet. Data were coded and then analyzed using Stata version 15. Internal consistency checks were carried out to ensure the validity and completeness of the data before the analysis. Descriptive statistics were reported for all sociodemographic, precautionary measures against COVID‐19, social attitude and adaptive coping strategies and health service utilization variables. *χ* ^2^ Tests were used for bivariate analysis. All variables were included in the binary logistic regression analysis to identify significant predictors of healthcare utilization. Backward (Wald) method was used with probability for removal at 0.1 for each step. Hosmer–Lemeshow goodness‐of‐fit test was conducted to examine goodness of fit of our final multivariable logistic regression model and variance inflation factor (VIF) was used to investigate multicollinearity. A VIF < 10 was considered acceptable. A *p* < 0.05 was considered to declare statistical significance.

### 2.5 Ethical approval

This study did not require ethical approval since it only included interactions involving survey procedures where the identity of the human subjects could not be readily ascertained, directly or through identifiers linked to the participants. We obtained web‐based informed consent from the participants after the disclosure of the objectives, anticipated risks, benefits, and confidentiality. The participants were not involved in the design or conduct or reporting or dissemination plans of the study. The methods were performed in accordance with relevant guidelines and regulations (Declaration of Helsinki).

## 3 RESULTS

### 3.1 Demographic characteristics of respondents

A total of 643 individuals participated in the survey (Table 1). More than two‐thirds of the respondents 422 (65.6%) were aged 18–30 years. Over half of the respondents 335 (52.1%) were females and more than half 407 (63.3%) had up to bachelor's degree level of education while 133 (20.7%) had up to masters or doctor of philosophy. The majority of the respondents 435 (67.7%) were single. In terms of religion, almost all respondents 611 (95%) were Christians. More than half 356 (55.4%) of the respondents were formally employed but less than half 297 (46.2%) earned 1000 GHS or below in a month while only 90 (14%) earned above 3000 GHS. More than two‐third 555 (86.3%) of the respondents were from a household of size 1–5. In all, 233 (36.2%) of the respondents reported not having a valid health insurance for healthcare (Table 1).

**Table 1:** Demographic characteristics of respondents.

Variables	Frequency (*N* = 643)	Percentage (%)
Age (years)		
18–30	422	65.63
Above 30 years	221	34.37
Sex		
Male	308	47.90
Female	335	52.10
Educational level		
Senior high/vocational or below	103	15.71
Bachelor degree/training college	407	63.30
Masters/PhD or its equivalent	133	20.69
Marital status		
Single	435	67.65
Married	208	32.35
Employment status		
Formally employed	356	55.37
Self‐employed	169	26.28
Unemployed	118	18.35
Religion		
Christianity	611	95.02
Islam	32	4.98
Average monthly income		
1000 GHS or below	297	46.19
Above 1000–2000 GHS	164	25.51
Above 2000–3000 GHS	92	14.30
Above 3000 GHS	90	14.00
Household size		
1–5	555	86.31
6 and above	88	13.68
Possession of valid medical insurance (medical insurance that has not expired)		
Doesn't have valid health insurance	233	36.24
Have valid health insurance cover	410	63.76

Abbreviations: GHS, Ghanaian Cedi; PhD, doctor of philosophy.

### 3.2 Public health preventive practices by respondents

Table 2 presents the results on the public health preventive practices during the first wave of the COVID‐19. The results showed that majority 590 (91.8%) of the respondents either always or most of the time covered their mouth when coughing or sneezing. Most 463 (72.0%) of the respondents also avoided sharing utensils such as plates, spoons, cups and forks during meal for fear of contracting COVID‐19 while almost all 607 (94.4%) respondents either always or most of the time washed their hands with soap under running water. Compared to wearing of face mask, 600 (93.3%) whenever going to public places, lesser proportion 79.5% (511) of respondents frequently used hand sanitizer to avoid contracting the COVID‐19 disease. After touching contaminated objects or surfaces, 587 (91.3%) of the respondents reported either washing their hands or sanitizing them.

**Table 2:** Public health preventive practices by respondents.

Variable	Always	Most of the time	Never	Occasionally	Sometime
Covering mouth when coughing and sneezing.	348 (54.12)	242 (37.64)	4 (0.62)	13 (2.02)	36 (5.60)
Avoiding sharing of utensils (e.g., plates, spoon, fork, cups) during meals.	279 (43.39)	184 (28.62)	54 (8.40)	47 (7.31)	79 (12.29)
Washing hands under running water with soap.	399 (62.05)	208 (32.35)	1 (0.16)	4 (0.62)	31 (4.82)
Sanitizing hands frequently to avoid contracting COVID‐19.	297 (46.19)	214 (33.28)	11 (1.71)	30 (4.67)	91 (14.15)
Wearing mask whenever going to public places.	421 (65.47)	179 (27.84)	1 (0.16)	6 (0.93)	36 (5.60)
Washing/sanitizing hands after touching contaminated objects.	409 (63.61)	178 (27.68)	2 (0.31)	10 (1.56)	44 (6.84)

Abbreviation: COVID‐19, coronavirus disease 2019.

### 3.3 Perceptions and coping strategies adopted in dealing with the pandemic

In terms of perceptions and behavioral changes adopted to dealing with the pandemic, more than half 357 (55.5%) agreed that unnecessary worry was created about the COVID‐19 pandemic (Table 3). More than one‐third 267 (41.5%) of the respondents stayed at home for 0–6 h a day while 170 (26.4%) and 127 (19.8%) stayed at home for 7–12 and 19–24 h a day, respectively to avoid contracting COVID‐19. Some 32 (5%) of the respondents reported drinking alcohol to help them get through the fear and anxiety caused by the pandemic while 19 (3%) took medicines for the fear/anxiety.

**Table 3:** Perceptions and coping strategies adopted in dealing with the COVID‐19 pandemic.

Variable	Frequency (*N*)	Percentage (%)
Too much unnecessary worry has been made about the COVID‐19 outbreak		
Strongly disagree	70	10.89
Disagree	139	21.62
Not sure	77	11.98
Agree	185	28.77
Strongly agree	172	26.75
Total	643	
Average number of hours staying at home per day to avoid COVID‐19		
0–6 h	267	41.52
7–12 h	170	26.44
13–18 h	79	12.29
19–24 h	127	19.75
Total	643	
I normally drink alcohol to help me get through the fear and/or anxiety cause		
Didn't drink alcohol	611	95.02
Drunk alcohol	32	4.98
Total	643	
I normally take drugs to help me get through the fear and/or anxiety cause		
Did take drug	624	97.05
Didn't take drug	19	2.95
Total	643	

Abbreviation: COVID‐19, coronavirus disease 2019.

### 3.4 Healthcare utilization

**Figure 1:**
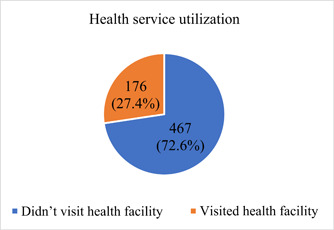
Healthcare utilization in Ghana during first wave of the pandemic.

**Figure 2:**
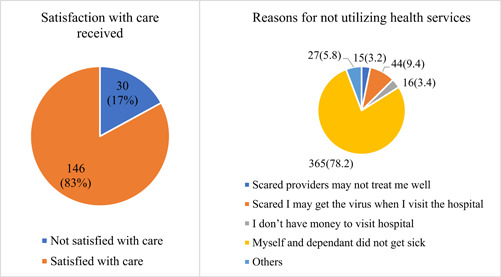
Level of satisfaction and reasons for nonutilization of healthcare.

A little above one‐fourth 176 (27.4%) of the respondents reported visiting a healthcare facility for healthcare during the first wave of the pandemic (Figure 1). Of those who utilized the healthcare, 30 (17%) reported they were not satisfied with the care provided them. While 44 (9.4%) reported not utilizing healthcare for fear of contracting the COVID‐19 disease, 365 (78.2%) reported they did not get sick during the period of the first wave (Figure 2).

### 3.5 Factors associated with healthcare utilization during first wave of the pandemic

The results showed that marital status of respondents (*χ* ^2^ = 9.23, *p* < 0.05), taking of medication to overcome the fear and anxiety created by the pandemic (*χ* ^2^ = 26.20, *p* < 0.05) and possession of valid health insurance (*χ* ^2^ = 4.70, *p* < 0.05) were statistically significantly associated with healthcare utilization (Table 4). The results further showed that respondents age 18–30 years 111 (63.1%) utilized health services more compared to those above 30 years 65 (36.9%). Similarly, respondents who were single 103 (58.5%) utilized healthcare more than the married. Also, healthcare was utilized more by females 97 (55.1%) compared to their male counterparts 79 (44.9%). Respondents who did not drink alcohol to overcome fear and anxiety created by COVID‐19, utilized healthcare 165 (93.8%) more compared to those who drank alcohol 11 (6.2%). Similarly, respondents who did not take medication to overcome fear and anxiety created by COVID‐19, utilized healthcare 161 (91.5%) more compared to those who took medication 15 (8.5%). It was observed that most of the respondents 124 (70.5%) who had valid health insurance utilized healthcare more than those without valid insurance 52 (29.5%).

**Table 4:** Factors influencing healthcare utilization using *χ* ^2^ test.

Characteristics	Utilized healthcare, *N* (%)	Not utilized healthcare, *N* (%)	*χ* ^2^	*p*‐Value
Age of respondents				
18–30	111 (63.07)	311 (66.60)	0.705	0.401
Above 30 years	65 (36.93)	156 (33.40)		
Total	176 (100.00)	467 (100.00)		
Marital status				
Married	73 (41.48)	135 (28.91)	9.228	0.002*
Single	103 (58.52)	332 (71.09)		
Total	176 (100.00)	467 (100.00)		
Sex of respondents				
Male	79 (44.89)	229 (49.04)	0.882	0.348
Female	97 (55.11)	238 (50.96)		
Total	176 (100.00)	467 (100.00)		
Employment status				
Formally employed	96 (54.55)	260 (55.67)	5.575	0.102
Self‐employed	55 (31.25)	114 (24.41)		
Unemployed	25 (14.20)	93 (19.91)		
Total	176 (100.00)	467 (100.00)		
Level of education				
Senior high/vocational or below	33 (18.75)	70 (14.99)	1.503	0.472
Bachelor degree/training college	106 (60.23)	301 (64.45)		
Masters/PhD or its equivalent	37 (21.02)	96 (20.56)		
Total	176 (100.00)	467 (100.00)		
Religion				
Christianity	161 (91.48)	450 (96.36)	6.444	0.011*
Islam	15 (8.52)	17 (3.64)		
Total	176 (100.00)	467 (100.00)		
Average monthly income				
1000 GHS or below	80 (45.45)	217 (46.47)	2.008	0.571
Above 1000–2000 GHS	40 (22.73)	124 (26.55)		
Above 2000–3000 GHS	27 (15.34)	65 (13.92)		
Above 3000 GHS	29 (16.48)	61 (13.06)		
Total	176 (100.00)	467 (100.00)		
Household size				
1–5	157 (89.20)	398 (85.22)	1.714	0.190
6 And above	19 (10.80)	69 (14.78)		
Total	176 (100.00)	467 (100.00)		
Whether respondent drunk alcohol				
Did not drink alcohol	165 (93.75)	446 (95.50)	0.831	0.362
Took alcohol	11 (6.25)	21 (4.50)		
Total	176 (100.00)	467 (100.00)		
Whether respondent took medications				
Didn't take medication	161 (91.48)	463 (99.14)	26.198	0.000*
Took medication	15 (8.52)	4 (0.86)		
Total	176 (100.00)	467 (100.00)		
Possession of valid medical insurance				
No valid insurance	52 (29.55)	181 (38.76)	4.695	0.030*
Valid insurance cover	124 (70.45)	286 (61.24)		
Total	988 (100.00)	712 (100.00)		

Abbreviations: GHS, Ghanaian Cedi; PhD, doctor of philosophy.

* Significance at 5% level.

### 3.6 Multiple logistic regression of factors influencing healthcare utilization in Ghana during first wave of COVID‐19 pandemic

Table 5 presents the multiple logistic regression of the factors that influence healthcare utilization in Ghana during the first wave of the COVID‐19 pandemic. After adjusting for the confounding effects of the variables of the sociodemographic characteristics, the remaining strong predictors of healthcare utilization include marital status of respondents (*p* < 0.05), religious affiliation (*p* < 0.05) and possession of valid medical insurance (*p* < 0.05). Compared to married respondents, singles were more likely to utilize healthcare. The odds of utilizing healthcare among respondents with valid health insurance was 1.5 times compared to the odds of utilizing healthcare among respondents without valid health insurance.

**Table 5:** Multiple logistic regression of factors influencing healthcare utilization in Ghana during first wave of COVID‐19 pandemic.

Characteristics	OR	95% CI	*p*‐Value
Age of respondents (years)			
18–30	Ref	Ref	0.517
Above 30 years	0.86	(0.537, 1.368)	
Sex			
Female	Ref	Ref	0.393
Male	0.84	(0.569, 1.247)	
Marital status			
Married	Ref	Ref	0.033^a^
Single	0.63	(0.409, 0.963)	
Religion			
Christianity	Ref	Ref	0.027^a^
Others	2.34	(1.102, 4.978)	
Educational level			
SHS or below	Ref	Ref	0.522
Bachelor degree	0.78	(0.462, 1.314)	
Master and above	0.69	(0.359, 1.327)	
Employment status			
Formally employed	Ref	Ref	0.344
Self‐employed	1.21	(0.779, 1.889)	
Unemployed	0.79	(0.0.439, 1.410)	
Household size			
1–5	Ref	Ref	0.293
6 And above	0.74	(0.421, 1.297)	
Average monthly income			
1000 GHS or below	Ref	Ref	0.526
Above 1000–2000 GHS	0.83	(0.505, 1.357)	
Above 2000–3000 GHS	1.23	(0.652, 2.305)	
Above 3000 GHS	1.26	(0.646, 2.471)	
Alcohol intake to overcome COVID‐19 fear and/or anxiety			
Didn't take alcohol	Ref	Ref	0.749
Took alcohol	1.14	(0.505, 2.585)	
Average number of hours staying at home per day to avoid COVID‐19			
0–6 h	Ref	Ref	0.209
13–18 h	1.33	(0.751, 2.354)	
19–24 h	0.84	(0.506, 1.401)	
7–12 h	0.71	(0.441, 1.144)	
Possession of valid health insurance			
No valid health insurance	Ref	Ref	0.040^a^
Valid health insurance cover	1.51	(1.020, 2.235)	

Abbreviations: CI, confidence interval; COVID‐19, coronavirus disease 2019; GHS, Ghanaian Cedi; OR, odds ratio; Ref, reference category; SHS, senior high school

^a^ Significant at 5% level.

### 3.7 Goodness‐of‐fit and multicollinearity examination

The results for the Hosmer–Lemeshow test with groups of 10 and 643 observations showed a *χ* ^2^ value of 14.10 and a *p*‐value of 0.08, suggesting that the model fits the data well. The multicollinearity test showed a minimum and maximum VIF values of 1.07 and 3.88, respectively with a mean VIF of 1.98, suggesting no evidence of multicollinearity.

## 4 DISCUSSION

### 4.1 Principal findings

This study examined self‐reported public health preventive practices, perceptions, and coping strategies and healthcare utilization and associated factors among the general public in Ghana during the first wave of the COVID‐19 pandemic. Majority (72.6%) of the respondents did not utilize healthcare during the first wave of the COVID‐19 pandemic. Compliance to the COVID‐19 public health preventive measures introduced in Ghana was high among the study participants. Critical factors identified to be significantly associated with healthcare utilization were marital status, religion, and possession of valid health insurance were statistically significantly associated with healthcare utilization.

### 4.2 Interpretation

The study found that only 27.4% of the respondents utilized healthcare during the first wave of the COVID‐19 pandemic. This resonates with the general findings on the global front of less healthcare utilization among the general public since the inception of the pandemic.^4^, ^5^, ^6^, ^7^, ^11^, ^12^ For instance, a similar study in Hong Kong which examined patterns of self‐reported health service utilization during the COVID‐19 outbreak found 30.4% of the public avoided medical consultations.^13^ Likewise, an assessment of healthcare utilization in a UK‐based privately insured population recorded as much as 70% reduction in non‐COVID care utilization among the population during the first wave of the pandemic.^11^ This demonstrates the major impact the pandemic had, particularly during the first wave. While this could be as a result of the imposed lockdowns and movement restriction policies during this phase of the pandemic, our findings showed that 78.2% of the respondents did not get sick during this period.

Moreover, all respondents surveyed in this study had some level of formal education which could influence their level of understanding health and implications of not maintaining good health. However, we found that 9% of respondents who did not utilize healthcare was because of fear of contracting the disease at the health facilities. This was not surprising at the time since there was either information overload or misinformation about the disease. Moreover, this could be because the health providers in Ghana at the time were not prepared.^12^ Afulani et al.^12^ found that only 27.8% of healthcare workers in public health facilities felt prepared to respond to the COVID‐19 pandemic.

Our findings showed that marital status, religion, and possession of valid health insurance were statistically significantly associated with healthcare utilization during the first wave of the pandemic. This confirmed earlier study where being married was found to be associated with avoiding medical consultation (healthcare utilization).^13^ Our findings further agreed with an earlier study which showed that health insurance coverage had a significant impact on the utilization of healthcare services during the COVID‐19 pandemic.^14^

We also found that overall compliance to the COVID‐19 public health preventive measures was high. The results showed that most of the respondents covered their mouth when coughing or sneezing (91.8%), washed their hands with soap under running water (94.4%), wore face mask whenever going to public places (93.3%) and sanitized their hands frequently (79.5%). The level of compliance observed in the current study was higher compared to those observed in India during the same period.^15^ In India, 91.8%, 79.1%, 45.4%, and 63.6% wore mask, covered nose and mouth, washed hands for at least 20 s and sanitized hands frequently with alcohol‐based hand sanitizer, respectively.^15^ This could be because the respondents in this current study were well informed with COVID‐19‐related knowledge as a result of their level of education compared to the earlier study conducted in India. Level of education was found to be positively associated with compliance with attitude and practice associated with COVID‐19.^16^ Moreover, the level of compliance observed in this study was higher than 70% reported in the southern African Kingdom of Eswatini.^17^

Additionally, the high level of compliance identified in this study was contrary to an earlier compliance audit of selected transportation stations in the Greater Accra region of Ghana.^18^ Affran et al.,^19^ found that hand washing practice was either not observed, or only infrequently even though majority (80%) of the stations had at least one Veronica Bucket with flowing water and soap while face masks were either not worn or only worn by a few passengers. While the authors attributed the lower level of compliance to the inadequate relevant public education about the importance of hand washing to prevent COVID‐19 infection coupled with a general low‐risk perception of COVID‐19 community spread and the cultural fact that people may not be used to washing their hands routinely in public, especially at the lorry stations, our study suggests that the higher level of compliance could be because of the higher educational level of the study respondents compared to those assessed by Affran et al.^19^ Moreover, at the time of our study, public health preventive practices were mandatory by the government of Ghana.^19^

However, we found that greater proportion of the respondents believed unnecessary worry and anxiety were created about the disease. The worry or anxiety reported reflected the general perceptions and experiences by many globally. For instance, in Nepal,^20^ reported that most participants in their study expressed distress and fear towards COVID‐19 and not only due to the perception that COVID‐19 is a highly fatal disease but also due to prejudice of uncertainty about the pandemic situation. However, the worry and anxiety created by the disease could to some extent contribute to the high level of compliance to the public health preventive measures observed in this study.

In relation to coping strategies, our results showed that most of the respondents stayed at home for more than 6 h in a day to prevent contracting the disease. However, in Nepal, reports indicated that the stay‐at‐home order was poorly complied with (40.2%).^21^ However, to overcome the fear and anxiety created by the pandemic, some respondents engaged in the intake of alcohol. The intake of alcohol to overcome the fear/stress created by the pandemic was also observed among United States adults in May 2020.^22^ This suggests a need for continuous public education using appropriate channels and language to ensure that COVID‐19 prevention information, advice, and recommendations are easily accessible and understandable by the general public.

### 4.3 Strengths of the study

The main strength of this study is that it provides new insights into the utilization of healthcare services and its predictors during COVID‐19 pandemic in Ghana.

### 4.4 Limitation of the study

This study has some limitations. First, the survey was online based. The sampling method could not avoid the bias of subjective selection since only those with access to internet and social media will have the opportunity to take part in the study. Thus, the findings might not apply to those without internet and social media platforms. The cross‐sectional nature of the study limits its ability to draw causal inference. Also, it is possible that the study might not have considered all possible risk factors that can predict healthcare utilization in this population.

## 5 CONCLUSION

There was generally high level of compliance with COVID‐19 public health preventive measures. However, there was low healthcare utilization coupled with fear of contracting the COVID‐19 disease at the health facilities and perception that unnecessary worry was created about the disease among the general public. The findings suggest the need for timely and effective public education using appropriate channels and language to ensure that future pandemics' prevention information, advice, and recommendations are easily accessible and understandable by the general public. These channels may include pandemic‐dedicated and easy‐to‐reach government social media platforms, websites, digital and traditional media as well as provision of government‐sanctioned population‐based short messaging services with appropriate and approved contents in both English and local languages and in the state that is easy to understand. This may help to counteract misinformation and its associated effects including the fear of contracting the disease at health facilities. Additionally, investment in health insurance coverage may contribute to healthcare utilization during future pandemics.

## AUTHOR CONTRIBUTIONS

**Dominic D. Gadeka**: Conceptualization; data curation; formal analysis; funding acquisition; investigation; methodology; project administration; resources; software; validation; visualization; writing—original draft; writing—review and editing. **Justice M. K. Aheto**: Data curation; formal analysis; investigation; methodology; project administration; resources; software; supervision; validation; visualization; writing—original draft; writing—review and editing.

## CONFLICT OF INTEREST STATEMENT

Justice M. K. Aheto is an Editorial Board member of Health Science Reports and coauthor of this article. He was excluded from editorial decision‐making related to the acceptance of this article for publication in the journal. All other authors declare no conflicts of interest.

## TRANSPARENCY STATEMENT

The lead author Dominic D. Gadeka affirms that this manuscript is an honest, accurate, and transparent account of the study being reported; that no important aspects of the study have been omitted; and that any discrepancies from the study as planned (and, if relevant, registered) have been explained.

## Data Availability

The datasets used and/or analyzed during the current study are available from the corresponding author on reasonable request.
